# Effectiveness of Gamification with a Narrative Adapted to the Player’s Profile in Obstetric Nursing Competencies: A Cluster Randomized Controlled Pilot Trial Protocol

**DOI:** 10.3390/nursrep16040104

**Published:** 2026-03-24

**Authors:** Sergio Mies-Padilla, Claudio-Alberto Rodríguez-Suárez, Aday Infante-Guedes, Héctor González-de la Torre

**Affiliations:** 1 Research Support Unit, Insular Maternal and Child University Hospital Complex, Canary Health Service, 35016 Las Palmas de Gran Canaria, Spain; 2 Faculty of Healthcare Science, University of Atlántico Medio (UNAM), 35017 Las Palmas de Gran Canaria, Spain; aday.infante@pdi.atlanticomedio.es; 3 Nursing Department, Faculty of Healthcare Science, University of Las Palmas de Gran Canaria (ULPGC), 35016 Las Palmas de Gran Canaria, Spain; hector.gonzalez@ulpgc.es

**Keywords:** gamification, clinical simulation, nursing education, obstetrics, personalized learning, player personality, artificial intelligence

## Abstract

**Background/Objectives**: Simulation-based education often lacks personalization, focusing on technical competence rather than individual student profiles. This protocol describes a study designed to evaluate whether adapting gamified narratives to nursing students’ personality profiles has the potential to support academic performance in obstetrics. This study aims to validate the integration of psychometric profiling and AI as a sustainable strategy for personalized clinical training. **Methods**: A cluster-randomized controlled longitudinal pilot trial will be conducted at the University of Atlántico Medio. The protocol has been submitted for registration at ClinicalTrials.gov (Registration Pending). Thirty-eight second-year nursing students meeting inclusion criteria (excluding repeaters or those with prior specialized training) will be assigned by natural practice to either a control group (generic gamification) or an experimental group (gamification adapted according to Player Personality and Dynamics Scale profiles using AI-generated content). The intervention comprises four clinical simulation sessions focusing on pregnancy and childbirth, which are managed via the Wix platform. The primary outcome is academic performance, measured as “Learning Gain” (post-test scores minus pre-test scores). Secondary outcomes include student satisfaction measured via the Gameful Experience Scale. Data will be analyzed using Mann–Whitney U tests to compare overall efficacy and intragroup evolution. To minimize observer bias, knowledge assessments will utilize automated, objective scoring, and participants will be blinded to the study hypothesis. **Expected Outcomes**: The study aims to establish the technical and pedagogical feasibility of integrating AI-adapted narratives into nursing curricula. It is anticipated that the personalized approach will show positive trends in learning gains and engagement patterns, providing a baseline for larger multicenter trials. **Conclusions**: This protocol presents a framework for “Precision Education” in nursing, shifting from “one-size-fits-all” simulations to student-centered adaptive training. The use of Generative AI makes such personalization sustainable and cost-effective for health science faculties.

## 1. Introduction

The use of simulation-based education to train health professionals in technical and non-technical skills has increased steadily in recent decades [1]. Within this framework, gamification has established itself as a new and rapidly growing educational methodology, especially in the academic field and in the continuous training of nurses and nursing students [2]. Gamification is widely defined as the use of game design elements in non-game contexts [3], functioning as a multidisciplinary process to improve motivation, engagement, and meaningful learning [4,5].

Gamification technologies, such as Escape Room games or immersive digital environments, involve introducing a team of players into a physical or digital space to solve puzzles in order to acquire professional skills in a complementary way to other methods [2,5]. This pedagogical approach is useful for increasing participants’ knowledge, improving their attitudes, promoting healthy behaviors, and reducing stress and anxiety [6]. In addition, it has been shown that the use of AI-driven adaptive educational games and gamified simulations promotes active learning, autonomy, and critical thinking, aligning with constructivist theories that center the learning process on the student [5,7], and can help overcome the dissonance between traditional methods and the demands of new generations of students [2]. Simulation has generally been applied successfully to improve outcomes in critical areas such as obstetric emergencies and neonatology, including reducing brachial plexus injuries and improving Apgar scores, with on-site multi-professional training demonstrating the best effect [8,9,10].

Despite the increasing adoption and demonstrated value of simulation and gamification in nursing and midwifery [11,12], there is marked heterogeneity in its implementation in different studies and programs, leading to disparities in assessment designs and tools [2,13,14]. In the context of simulation-based education, the lack of global consensus on how to assess or integrate skills is reflected in the fact that curricula are often based on the number of cases completed rather than objective outcomes [13,15]. In addition, the efficacy of educational interventions is intrinsically linked to specific characteristics such as the content, “dose”, and optimal methodology, aspects that still require identification through experimental studies [9,16]. There is a risk of the “novelty effect”, where initial motivation may decrease after the first few weeks if the challenge is not maintained or if the content is not properly adapted [17].

The lack of a personalized approach means that training often focuses on technical competence without fully integrating interpersonal skills and factors from the clinical setting, such as communication with patients and peers [15,18]. Consequently, existing programs do not always adapt optimally to individual student needs or facilitate long-term skill retention, as most studies do not perform deferred post-intervention measurements to ensure the permanence of acquired knowledge [2].

Despite the theoretical consensus on the importance of personalization, there is a notable paucity of controlled empirical studies that directly compare generic gamification and adapted gamification, with more studies [19,20] investigating gamification versus non-gamification. Personality traits fundamentally shape student engagement, intrinsic motivation, and responsiveness to gamified interventions, as different player typologies react differently to specific game mechanics. To address the need for personalization and homogenization in gamification-based training, this study proposes the implementation of an adaptive model integrated with standardized assessment tools. Specifically, the Player Personality and Dynamics Scale (PPDS), validated in Spanish, will be used to identify player profiles in educational gamification contexts with narrative elements [21]. While various classifications of player profiles exist in the literature, the PPDS was specifically selected for this protocol because it is already adapted and psychometrically validated in Spanish. Applying non-adapted frameworks to our specific student cohort could introduce linguistic and cultural biases, making the PPDS the most methodologically rigorous choice available to ensure measurement accuracy in this context.

These adaptive models are crucial because evidence suggests that the most effective interventions are those that include multiple components and can tailor the narrative to individual needs [22,23]. In healthcare education, the incorporation of advanced high-fidelity simulators with structured feedback mechanisms and continuous assessment is vital to refining technique and fostering clinical decision-making skills [24]. Recent innovations include using artificial intelligence for adaptive responses in simulation environments, indicating a trend towards personalized and sustainable competency-based training strategies [25]. This paradigm shift aligns directly with the emerging concept of ‘Precision Medical Education’, which emphasizes the use of technology and continuous data streams to tailor educational trajectories to the specific needs of each learner [26,27]. Employing an approach that combines individualized measurement, through the PPDS [21], with adaptability enables the design of personalized gamified experiences that improve learning and interaction in the classroom, optimizing professionals’ ability to acquire the necessary competencies [14] and improve their quality of care in clinical practice.

Current research has reached a saturation point regarding the general efficacy of gamification versus traditional methods, yet a critical gap remains in understanding how to optimize these interventions for diverse types of learners. Generic gamification carries inherent risks: if the game mechanics do not align with the student’s intrinsic motivation, it can lead to frustration, cognitive overload, or disengagement, potentially mitigating the educational benefits.

This study is necessary and timely because the recent maturation of Generative Artificial Intelligence (AI) finally enables scalable and sustainable personalization of clinical narratives—a feat previously considered too resource-intensive for standard curricula. The novel and testable contribution of this protocol is the shift from a binary comparison (game vs. no game) to a nuanced analysis of mechanics–profile fit. Therefore, generic gamification was chosen as the active comparator to isolate and quantify the specific added value of the ‘personalization’ variable.

Consequently, the aim of this study is to evaluate the effectiveness of a profile-adapted gamified intervention compared to a generic gamified intervention in improving the academic performance of nursing students regarding obstetric competencies upon completion of the four-week program. Based on this aim, the following hypotheses are proposed:

- Primary hypothesis—the Experimental Group (EG), with exposure to the profile-adapted narrative, will demonstrate a significantly higher global Learning Gain (LG) compared to the Control Group (CG) receiving generic gamification.
- Secondary hypothesis—there is a positive correlation between the level of student satisfaction and academic performance (LG) regardless of the group.

## 2. Materials and Methods

### 2.1. Administrative Information

This manuscript represents Protocol Version 1.0, dated 16 January 2026. The primary sponsor for this study is the University of Atlántico Medio. The sponsor had no role in the design of this study and will not have any role during its execution, analysis, interpretation of the data, or decision to submit results. The protocol has been submitted for registration to ClinicalTrials.gov. The registration is currently pending validation.

### 2.2. Study Design

**Figure 1:**
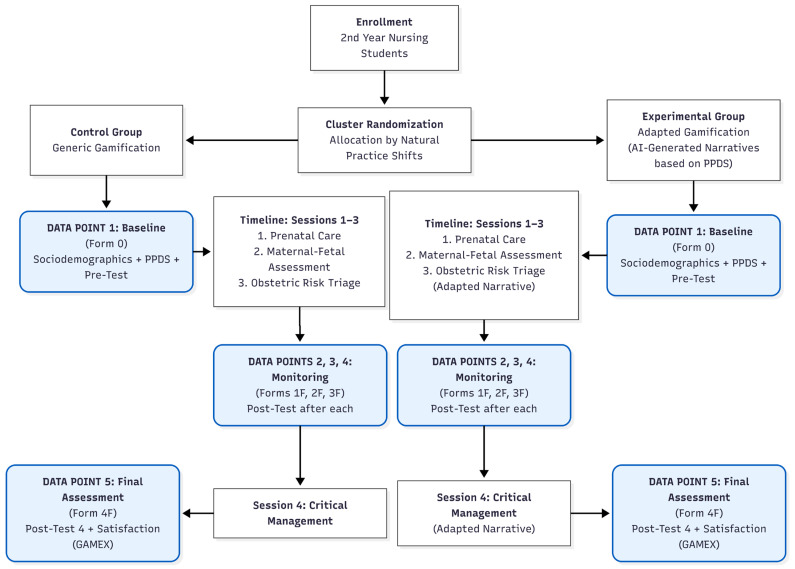
Schedule of enrollment, interventions, and assessments.

A cluster-randomized, prospective, comparative, and longitudinal pilot trial with two parallel groups is proposed. The study employs a 1:1 allocation ratio (one cluster per arm) and follows a superiority framework regarding the primary hypothesis. The CG will receive a generic gamified intervention, and the EG will receive an adapted gamified intervention. The study timeline spans four consecutive weeks. Participant flow, from enrollment to close-out, follows the schedule of assessment detailed in Figure 1.

The research questions and outcome measures were developed by the teaching faculty based on curricular requirements. Students were not involved in the design, recruitment, or conduct of this study. However, results will be disseminated to participants upon completion of the academic year.

### 2.3. Study Scope and Population

This single-center study will be carried out in an academic setting at the Faculty of Health Sciences of the University of the Atlántico Medio (Las Palmas de Gran Canaria, Spain) during the second semester of the 2025–2026 academic year. The target population will be second-year students of the bachelor’s degree in nursing enrolled in the course “Reproductive Health Nursing and Sex Education”.

The inclusion criteria are as follows: active enrollment, face-to-face attendance, and technological access. Students repeating the course, students with prior specialized training, and students with uncorrected sensory impairments will be excluded. The criteria for withdrawal once the study begins include incomplete data, abandonment of the session before the simulation is concluded, technological failure, or detected cross-contamination.

### 2.4. Sample Size

As this is a pilot feasibility study, formal a priori power analysis was not conducted. The sample size of 38 students represents a convenience sample limited by the ecological environment of the classroom. Therefore, hypothesis testing in this protocol is not intended to provide confirmatory evidence of superiority, but rather to calculate the preliminary effect size and variance to inform the power calculation for future, fully powered multicenter trials.

### 2.5. Randomization and Masking

Given the nature of practical teaching (in which groups are pre-established by enrollment), simple individual randomization is not possible. Therefore, a cluster randomization design will be implemented. An independent researcher not involved in the teaching process will use (RANDOM.ORG; Randomness and Integrity Services Ltd., Dublin, Ireland) computerized random number generation software to assign the practice shifts (clusters) to either the CG or EG arm. Allocation concealment is ensured as the assignment will only be revealed to the Principal Investigator and students on the first day of the intervention.

Given the highly experiential nature of the intervention, participant blinding to the educational differences between conditions is likely only partial. However, participants will remain naive to the comparative study hypothesis and their specific allocation to either the control or experimental cohort. However, due to the logistical requirements of the intervention (the distribution of specific login codes), the Principal Investigator (PI) cannot be blinded during the sessions. To mitigate bias and ensure external control, statistical data analysis will not be performed by the PI. Instead, it will be performed by a different researcher (the co-author) who was not involved in the teaching process, using a dataset in which the group assignment is masked (coded as Group A vs. Group B).

To maintain this blinding and prevent the participants from distinguishing between the narrative formats (generic vs. adapted), the intervention relies on the physical and temporal separation of the clusters. Since Group A and Group B attend consecutive but distinct shifts, there is no simultaneous visual contact with the screens of the Control and Experimental groups. Furthermore, the clinical content and the structure of the Google Forms (Google LLC, Mountain View, CA, USA) remain visually identical for both groups; only the Wix interface ‘wrapping’ differs, and this is not visible to the other group due to the shift separation.

### 2.6. Recruitment Strategies

Recruitment will be conducted using a saturation strategy of the available academic cohort. Potential participants will be approached during the introductory briefing of the first scheduled session. Due to the academic nature of the setting, the recruitment period is restricted to this initial contact point to ensure all students start the intervention simultaneously.

The PI will present the study objectives and the “Patient Information Sheet” to the entire class. To mitigate the potential for coercion given the teacher-researcher dual role, the recruitment protocol strictly differentiates between educational participation and research participation: all students will engage in the gamified sessions as part of their standard curricular training; only the data of those students who voluntarily sign the Informed Consent will be processed for analysis; no financial incentives will be offered. Participants in the CG are guaranteed access to the experimental platform after the study concludes (post-Session 4) to ensure equitable learning opportunities.

### 2.7. Interventions

The intervention will be carried out in the nursing classroom of the Faculty of Health Sciences. It will be structured in four face-to-face sessions lasting 2 h each. The process will be led entirely by the PI, who will act as a teacher, clinical facilitator, and administrator of the gamified platform.

Prior to the start of the study, the digital learning environment will be developed: narrative scripts will be generated using generative AI tools. It is important to note that Generative AI is used exclusively offline during the pre-intervention design phase as a drafting tool for the narrative scripts. The AI does not interact with students in real-time, nor does it evaluate their clinical answers. All AI-generated texts are thoroughly reviewed, clinically curated, and validated by the PI prior to their integration into the Wix platform (Wix.com Ltd., Tel Aviv, Israel) to prevent any clinical hallucinations or biases. In the CG, the narrative will be linear and generic for each topic. In the EG, there will be five narrative variations per theme, adapted to the profiles of the PPDS (Tryhard, Coacher, Esthetic, Joker, and Toxic). To clarify the specific elements of adaptation and ensure reproducibility, Table 1 details the differences between the generic and adapted narratives. It is important to note that the level of clinical difficulty and the learning objectives remain invariant between groups to ensure the validity of the academic performance comparison: patient vital signs, clinical history, learning objectives, and multiple-choice questions are identical in both groups. The adaptation focuses exclusively on the narrative ‘wrapping’, the tone of the feedback, and the motivational mechanics (gamification elements) triggered by the Wix platform.

A master website will be set up on Wix. Progress will be managed through “Digital Locks”, where students will be required to solve clinical issues embedded in Google Forms. Upon submitting the correct answer, the form will return a password that will allow the student to unlock the next page (level) in Wix.

The total cohort of 38 students will be divided into two consecutive practice shifts (Group A and Group B): one with 18 students and the other with 20. In the first session, software will be used to randomly assign which group will have the first shift. Students will remain in their assigned group for all four sessions to avoid cross-contamination. The program consists of four compulsory thematic modules:

- Session 1: Prenatal care;
- Session 2: Maternal-fetal assessment and well-being;
- Session 3: Obstetric risk triage;
- Session 4: Critical management in pregnancy.

**Figure 2:**
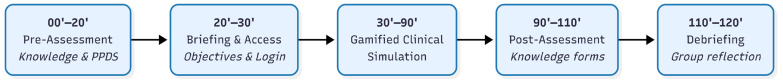
Schematic diagram of the daily 120 min session workflow.

Each day of the internship follows the same 120 min (2 h) time structure, which is visually summarized in Figure 2.

During the middle 60 min (intervention), the PI will monitor activity as the students interact with their devices. In the CG, all learners receive the same login URL and password, and the storytelling in Wix is standard. The mechanics are the same for everyone: progress bars are present, and points are awarded for correct answers. In the EG (PPDS-Adapted Gamification), at the beginning of each session, the PI provides differentiated codes (according to role) based on the learner’s profile to access five parallel narratives in Wix for the same clinical case. The clinical goal is the same for all participants, but the narrative “wrapping” and side mission change.

To ensure fidelity, the PI will be the only person with admin privileges in Wix and access to the Google Forms answers in real-time. Attendance will be monitored using signatures. The PI will resolve technical or clinical questions during the session, ensuring that the password lock in Wix does not impede the progress of learning.

Forms will be set up as “required steps” within the Wix narrative. In the case of the EG, the knowledge forms will be identical in content but esthetically matched to the identified player’s profile.

### 2.8. Outcome Variables

The main outcome variable will be academic performance, LG, understood to reflect new knowledge acquired on the subject under study. The degree of assimilation of the theoretical and practical concepts of pregnancy and childbirth in each of the four clinical cases will be evaluated. To this aim, an “ad hoc” questionnaire comprising 40 multiple-choice questions was designed by the PI, who is a certified Nurse Specialist in Obstetrics and Gynecology (Midwife) and the lecturer in charge of the class. The items were constructed to strictly align with the specific curricular contents and learning objectives of the ‘Reproductive Health’ syllabus. To ensure external content validity prior to administration, this initial draft will be reviewed by an external panel of five experts (three academic midwives and two obstetricians). Regarding reliability, since this is a pilot study, internal consistency and item analysis will be calculated post hoc to validate the instrument for future trials. The questions in the final questionnaires (administered post-simulation at each session) will address the same content as the initial questionnaire, but the form and order of the answers will be modified. Learning gain will be calculated using a simple difference (post-test score minus pre-test score), normalizing the results on a scale of 0 to 10 points. An arithmetic mean of the learning gain scores obtained from the four use cases will be calculated.

The secondary outcome variables will be

- Satisfaction: The students’ subjective perception of the usefulness, fun, and motivation generated by the activity will be evaluated using the Gameful Experience Scale (GAMEX) [28]. This validated 27-item instrument in Spanish is suitable for measuring game experience in nursing students’ training. It uses Likert-type responses to measure the multidimensional nature of the gamified experience in the educational context. The six dimensions include enjoyment, abstraction, creative thinking, activation, absence of negative effects, and mastery. This model demonstrated high overall reliability, with a Cronbach’s alpha of 0.85. The scoring format uses a five-point Likert scale. In the proposed study, measurement will be conducted exclusively at the end of the intervention (Session 4) to capture the students’ overall perception of the program. Prior to analysis, items with negative wording (reverse punctuation) will be recoded so that higher scores consistently reflect a more positive experience. The statistical report will present results for both the total aggregate score and the independent mean scores for each of the six dimensions.
- Player Profile: the students’ psychological and behavioral predisposition to game dynamics and narratives will be assessed using the PPDS in its validated Spanish version [21]. The PPDS is a psychometrically validated instrument composed of 21 items rated on a six-point Likert scale specifically designed to identify player profiles in the context of educational gamification based on narratives to measure the frequency of certain behaviors, where 1 means “Never” and 6 means “Always”. After a student completes the questionnaire, the system does not place them in a single category but generates five independent scores, one for each profile, showing which of the following profiles they align with the most: Tryhard (achievement- and competency-oriented); Coacher (oriented toward leadership and help); Esthetic (narrative and visual beauty-oriented); Joker (oriented to humor and surprise); or Toxic (a technical gamification term referring to players oriented toward testing system limits, seeking loopholes, or disrupting boundaries, often associated with lateral thinking rather than a negative personal judgment). The scale demonstrated high overall reliability (omega = 0.93).
- Gamification Experience: Previous experience in gamification will be evaluated using the following question: Have you participated in similar activities before? The students can respond “yes” or “no”.
- Sociodemographic variables: Age is understood as the time that a person has lived, counting from birth to the time of the survey, measured in whole years; gender is understood as a variable that refers to the social and cultural construction that defines the roles, behaviors, activities, and attributes that a society considers appropriate, with the answer options male, female, and non-binary; and the average grade considered to mean the average grade achieved in the first year of the nursing program to monitor the differences between the groups.
- Feasibility and implementation outcomes: given the pilot nature of this trial, the following feasibility metrics will be formally tracked to inform the design of the subsequent definitive trial: recruitment rate, retention rate, technical failure rate, time-on-task compliance, and qualitative evidence of cross-contamination.

### 2.9. Data Collection Method

The study employs a hybrid data collection strategy to clearly separate the educational game mechanics from the research evaluation process. All formal assessments related to the study outcomes—including sociodemographic data, the PPDS, and the knowledge tests—will be administered exclusively in physical paper format. This ensures that research data collection does not depend on the stability of the digital platform during the session. Google Forms will be used solely as a functional component of the gamification (acting as “digital locks”). Participants enter their clinical answers into these forms to obtain the passwords required to advance through the Wix narrative. These digital responses serve an instructional purpose and are not the primary source for academic performance analysis.

To ensure high-quality data and minimize errors during the transition from paper to digital analysis, each student will be assigned a unique alphanumeric code on their first paper form (Form 0), which must be manually transcribed by the student onto all subsequent paper assessments (Forms 1F to 4F) to enable longitudinal matching. The PI will manually enter the data from the paper forms into a pre-configured offline database. To promote data integrity, a random 10% sample of the paper forms will be cross-checked against the digital database by a co-author to ensure transcription accuracy.

The primary strategy for participant retention is the integration of the trial into the mandatory face-to-face academic calendar. The educational value of the simulations as preparation for the final exam serves as the main incentive for completion. Reasons for any missing sessions or incomplete paper forms will be recorded in the field diary to be reported as feasibility outcomes.

### 2.10. Data Analysis

This publication constitutes the full trial protocol and detailed statistical analysis plan. No separate analysis plan document exists. Any future deviations from this plan will be reported and justified in the final results publication.

Data analysis will be carried out using the Jamovi 2.6.44 statistical package (The jamovi project, Sydney, Australia). Statistical significance level of *p* < 0.05 will be established; however, given the exploratory nature of these analyses, we will rely less strongly on *p*-values and give greater importance to effect sizes and 95% confidence intervals. In addition to statistical significance testing, the effect size and 95% confidence intervals will be reported for all primary outcomes. This approach prioritizes the estimation of the magnitude of the intervention effect, which is the primary statistical goal of this pilot study.

Before inferential analysis, the raw variables extracted from the forms 1F to 4F will be processed to generate the result metrics. The absolute LG will be calculated using the following formula: (PostTestScore) − (PreTestScore). To facilitate comparison, raw scores will be normalized to a standard grading scale of 0 to 10 points. This calculation will be applied consistently across all four sessions. To handle missing data, given the compulsory attendance requirement, a complete-case analysis approach will be utilized; participants with incomplete data for a session will be excluded from that specific analysis. Consequently, if a participant misses a session, their global LG metric will be calculated as the mean of their completed sessions only.

To handle the repeated measures design within the constraints of the pilot sample size, the primary analysis will not utilize complex mixed-model ANOVAs. Instead, the LG scores from the four sessions will be averaged to generate a global LG metric for each student. This summary measure is educationally meaningful as it reflects the overall assimilation of the obstetric module, mitigating the impact of performance fluctuations in any single session. This aggregation strategy aims to reduce intra-individual variance and provide a more robust estimator for the main group comparison. The normality of the dependent variables will be evaluated using the Shapiro–Wilk test. Given the estimated sample size, the use of non-parametric statistics is assumed to be a priority, unless the data demonstrates a normal distribution.

A descriptive analysis of sociodemographic data and previous experience will be carried out, qualitative variables will be presented as absolute and relative frequencies, and quantitative variables will be presented as medians and interquartile ranges (if the distribution is not normal) or means and standard deviations if the distribution is normal.

To compare the overall efficacy (CG versus EG), the homogeneity at baseline will be checked. The Pre-test (Form 0) scores and the average grades according to academic records will be compared between both groups using the Mann–Whitney U test to ensure that the groups are comparable despite the cluster randomization. Additionally, prior gamification exposure and the distribution of PPDS profiles will be descriptively compared at baseline to identify any major initial imbalances between the clusters

To test the primary hypothesis, the mean global LG of the four sessions between the CG and the EG will be compared using the Mann–Whitney U test for independent samples. Given that the study design involves two natural clusters of unequal size (n = 18 and n = 20), the main hypothesis test will be performed at the participant level. Given the limitation of having only one cluster per arm, inferential statistics will be considered strictly exploratory, and results must be interpreted with caution.

Regarding the secondary outcomes, the analysis of the five PPDS profiles will be strictly descriptive and exploratory, given the limited sample size. Subdividing the experimental group (n = 20) into five profile categories yields cell sizes too small for robust inferential statistics; hence this approach prioritizes statistical rigor by avoiding underpowered hypothesis testing on these specific sub-variables.

To analyze the students’ perception at the end of the study, the satisfaction scores (GAMEX) will be compared between groups using the Mann–Whitney U test. Finally, an exploratory Spearman correlation analysis will be performed between satisfaction and LG to determine if the students who enjoyed the experience the most were also those who learned the most.

Additionally, exploratory analyses will be conducted to examine the potential influence of sociodemographic factors on the intervention outcomes. Spearman’s rank correlations will be used to assess the relationship between age, prior academic grades, and the global LG. Gender differences regarding performance and satisfaction will also be descriptively explored. However, due to the limited pilot sample size, these specific sociodemographic subgroup analyses will be treated strictly as hypothesis-generating.

### 2.11. Data Protection and Trial Management

To ensure anonymity and facilitate longitudinal data matching, a double-layer security protocol will be applied. A third-party platform, Google Forms, will be used under the PI’s license solely as a data entry interface, while the intervention will be hosted on Wix. Crucially, user registration with personal emails will not be required on Wix; access will be facilitated using generic passwords or role codes provided by the teacher.

Each student will be assigned a unique alphanumeric code to allow for traceability without revealing personal identities in the final statistical matrix. The master database containing the link between the code and the participant’s identity will be stored locally and guarded exclusively by the PI on a device with encrypted storage and protected by double-factor authentication. This master file will be destroyed once variable cross-referencing is complete. Due to the short duration and educational context of the pilot, interim analyses are not planned.

Given that the PI also teaches the subject, the analysis of the research data will be carried out strictly after the official transcripts of the participating students have been finalized and locked. This temporal separation ensures that the students’ performance in the “game” cannot bias their official grade.

Given the educational nature of the intervention, the risk of physical harm is negligible. However, specific protocols have been established to mitigate potential psychological, technical, and ethical risks. To ensure clinical and ethical safety, all narratives and clinical cases generated with the assistance of Generative Artificial Intelligence will be reviewed, corrected, and validated by the PI. This process guarantees that the content is free from gender bias, racial stereotypes, or erroneous clinical recommendations (hallucinations). To prevent potential offense or stigmatization arising from the PPDS profiles, students will be explicitly informed during the briefing that these categories are strictly playful archetypes for game mechanics and do not constitute a psychological or clinical diagnosis. In the event of persistent technical failures, the session will immediately revert to a traditional seminar format to prevent student anxiety or loss of learning time. The PI will maintain a “Field Diary” to record any spontaneous reports of student distress, technical incidents, or expressions of dissatisfaction. Any event requiring the interruption of a student’s participation will be reported to the Ethics Committee.

## 3. Expected Outcomes

As a study protocol, this article does not report empirical findings. However, based on the proposed design, the study is anticipated to generate data in the following categories:

- Feasibility and implementation: The primary outcome is to establish the technical and pedagogical feasibility of integrating AI-generated narratives into the standard nursing curriculum. We expect to report completion rates, time-on-task data, and technical bottlenecks identified in the Wix platform.
- Academic performance trends: Regarding the LG, the study will provide preliminary estimates of the effect size comparing the adapted (EG) versus generic (CG) methodologies. Rather than confirming statistical superiority, the data will determine if the personalized approach shows a positive trend that justifies the calculation of a larger sample size for future randomized clinical trials.
- Engagement patterns: Through the exploratory analysis of the PPDS profiles, we anticipate characterizing how different personality types interact with the gamified elements. We expect to describe whether specific profiles show differential satisfaction scores compared to the standard user, providing a baseline for tailored educational strategies.
- Correlation data: We expect to obtain statistical evidence regarding the relationship between student satisfaction (GAMEX scores) and academic performance, clarifying whether higher engagement correlates with actual knowledge retention in this specific demographic.

## 4. Discussion

This protocol describes a pilot intervention designed to overcome the “one-size-fits-all” limitation in current simulation-based education. Although the literature supports the efficacy of gamification, there is a lack of reproducible methods to adapt these narratives to diverse student personalities on a large scale. The significance of this study lies in its use of Generative AI to make narrative personalization feasible and sustainable for standard nursing curricula, addressing the need for cost-effective implementation strategies. If successful, this protocol could serve as a blueprint for “Precision Education” [27] demonstrating how technology can drive the next era of personalized assessment [26]. In this model, training methodologies are tailored to the psychometric profile of the learner to maximize engagement and retention.

The necessity for such an adapted approach is underscored when comparing our proposed framework with the challenges highlighted in the recent literature. For instance, while studies [17] confirm that gamification generally enhances short-term motivation, they also warn of the ‘novelty effect’, where engagement drops if the digital environment fails to sustain a meaningful connection with the learner. Generic implementations often struggle with this, as a leaderboard that motivates a competitive student might actively demoralize a collaborative one. Furthermore, most contemporary research [20] still focuses on binary comparisons (gamification versus traditional lectures). By using generic gamification as the active control group, our protocol aims to move the conversation beyond mere technological implementation toward true precision education, isolating the specific pedagogical value of psychological profiling.

The choice of a cluster-randomized design was driven by ecological validity, with the aim of testing the intervention in a real-world classroom setting rather than a controlled laboratory. However, this introduces specific risks—primarily the ethical conflict of the dual role of the Principal Investigator as both researcher and teacher. As detailed in Section 2.5 and Section 2.11, this risk is mitigated by separating the research data collection from the academic grading process and ensuring that the statistical analysis is blinded and performed by an independent co-author. Furthermore, the risk of “contamination” between groups is addressed by strictly separating the sessions and using rotating passwords for the digital platform.

### 4.1. Limitations

A methodological limitation that is worth highlighting is the size of the sample, which is limited by the official ratio of students per internship group and could reduce the statistical power of the study, making it difficult to detect significant small differences between subgroups. To mitigate this issue, the use of non-parametric tests will be prioritized. In addition, the study is planned as a pilot, whose main objective is to validate the statistical trend and effect size for future multicenter trials.

Regarding the randomization of entire natural groups rather than individuals, there is a risk that the groups may not be entirely homogeneous at baseline. Specifically, having only one cluster per arm constitutes a major design limitation, as the intervention effect may be confounded with cluster-level differences. Therefore, any efficacy comparisons between groups in this pilot phase are strictly exploratory. To address this, a strict statistical verification of comparability will be conducted. If significant baseline differences are detected, they will be explicitly reported and considered potential confounding variables during the interpretation of results, acknowledging that parametric adjustment methods may not be applicable given the sample size constraints.

It is important to acknowledge that the assessment of academic performance acquisition in this study is restricted to the cognitive domain (knowledge and theoretical decision-making), which will be measured via written tests. Due to the pilot nature of the study and logistical constraints, objective measures of psychomotor clinical competence or behavioral transfer to practice, such as Objective Structured Clinical Examinations or checklists in high-fidelity environments, will not be included. Future phases of this project will aim to incorporate these practical dimensions to evaluate the ‘transfer of training’ to the bedside.

It should be noted that there may be a “Novelty Effect” and the improvement in initial performance may simply be due to the novelty of using technology (Wix) in class and not to the specific narrative adaptation. The longitudinal design of four sessions (spanning four consecutive weeks) will help to dilute this effect. By monitoring the students over a one-month period, which corresponds to the ‘first few weeks’ where initial motivation typically decreases, we can determine if engagement is maintained until the final session.

As the researcher is also the teacher who evaluates the coursework, this could introduce unconscious bias (Pygmalion Effect) to favor the EG or generate in the students a desire to please the teacher by responding positively to the surveys. To address this situation, the knowledge-assessing questionnaires contain multiple-choice questions to facilitate automatic and objective correction and eliminate subjectivity on behalf of the evaluator. Anonymity will be guaranteed for the satisfaction surveys to encourage honesty.

As for the profile self-evaluation through the PPDS, the classification of the students depends on their sincerity when answering the test. It may be that a “Toxic” student does not recognize themselves as such and marks “Coacher” answers due to social desirability, thus being assigned a narrative that does not correspond accurately to their profile. This issue will be addressed by clearly explaining that there are no “good or bad” profiles, thus reducing social pressure.

Another limiting aspect is the risk of contamination between groups. As students are classmates from the same faculty, the CG students may learn about the existence of the EG’s “special missions” and feel demotivated (demoralizing effect) or try to obtain the EG passwords. To address this issue, Wix login passwords will be changed on a per-session and group basis, and explicit confidentiality will be required as part of the “game contract”. To actively monitor this risk without increasing survey fatigue, the Principal Investigator will utilize the pre-established “field diary” to record any spontaneous student disclosures or observable evidence of cross-contamination between the shifts. Any detected contamination will be reported as a feasibility metric and factored into the interpretation of the exploratory efficacy results. While this observational approach is suitable for a pilot, future definitive trials will benefit from incorporating a formal self-report question to empirically measure cross-contamination rates.

Finally, as a logistical limitation, and given the nature of the study, failures in the faculty’s Wi-Fi connection, Wix server crashes, or problems with students’ mobile devices could interrupt the intervention. An alternative plan will be available, with changes in the teaching schedule agreed upon with the students so that a new practical session may be scheduled.

### 4.2. Dissemination Plan

The results of this pilot study will be disseminated through publication in high-impact, peer-reviewed journals in the fields of nursing education and medical informatics and presented at international conferences. Authorship eligibility will be determined according to the International Committee of Medical Journal Editors guidelines. A summary of the key findings, written in plain language, will be sent to all participants upon study completion.

Regardless of the statistical significance of the results, the de-identified individual participant data, the data dictionary, the statistical analysis code, and the specific AI prompts used to generate the narratives will be deposited in a public open-access repository upon publication. This ensures full transparency and allows for independent replication of the study findings.

## 5. Conclusions

This protocol presents a structured methodological framework to address the lack of personalization in simulation-based nursing education. The primary contribution of this study will be to establish the technical and pedagogical feasibility of using Generative AI to create profile-adapted clinical narratives, a process that has historically been resource-prohibitive for standard curricula.

Rather than assuming the superiority of the adapted method, this pilot study aims to provide the first empirical evidence regarding the relationship between player personality (PPDS profiles) and engagement with digital clinical cases. The data obtained will be critical to determining whether a “precision education” approach yields sufficient improvements in learning gains or satisfaction to warrant implementation in larger, randomized clinical trials.

Ultimately, the successful execution of this protocol will validate a scalable workflow for integrating psychometric profiling into the Learning Management Systems of health science faculties, potentially shifting the paradigm from “one-size-fits-all” simulations to student-centered adaptive training. By doing so, this research provides a novel and cost-effective blueprint to advance precision education in the nursing field.

## Figures and Tables

**Table 1:** Comparison of gamification elements between control and experimental groups.

Element	Control Group (Generic)	Experimental Group (Adapted to Profile)
Clinical Difficulty	Invariant. Identical clinical cases and complexity.	Invariant. Identical clinical cases and complexity.
Narrative Tone	Neutral, academic, and linear.	Adapted, e.g., “Epic/Heroic” for Tryhard; “Humorous/Satirical” for Joker; “Emotional/Empathetic” for Socializer.
Visual Esthetics	Standard medical imagery (clean, white/blue).	Adapted, e.g., “High-fantasy/Dark” for Esthetic; “Leaderboards/Badges” prominence for Tryhard.
Feedback Style	Correct/incorrect + standard explanation.	Adapted, e.g., “Challenge failed, try again!” (Tryhard) vs. “Don’t worry, let’s learn together” (Coacher).
Side Missions	None.	Specific: Hidden “Easter eggs” (Joker), esthetic puzzles (Esthetic), or speed challenges (Tryhard).

## Data Availability

The data relating to the implementation of the intervention and the results derived from this research will be available upon justified request to the corresponding authors.

## Public Involvement Statement

No public involvement in any aspect of this research.

## Guidelines and Standards Statement

The study protocol has been developed in adherence to the Standard Protocol Items: Recommendations for Interventional Trials (SPIRIT) 2025 statement.

## Abbreviations

The following abbreviations are used in this manuscript:

AI	Artificial Intelligence
CG	Control Group
EG	Experimental Group
GAMEX	Gamified Experience Scale
LG	Learning Gain
PI	Principal Investigator
